# Involvement of CX3CL1/CX3CR1 Signaling in Spinal Long Term Potentiation

**DOI:** 10.1371/journal.pone.0118842

**Published:** 2015-03-13

**Authors:** Chao Bian, Zhi-Qi Zhao, Yu-Qiu Zhang, Ning Lü

**Affiliations:** Institute of Neurobiology, Institutes of Brain Science and State Key Laboratory of Medical Neurobiology, Collaborative Innovation Center for Brain Science, Fudan University, Shanghai, 200032, China; Toronto Western Hospital, CANADA

## Abstract

The long-term potentiation (LTP) of spinal C-fiber-evoked field potentials is considered as a fundamental mechanism of central sensitization in the spinal cord. Accumulating evidence has showed the contribution of spinal microglia to spinal LTP and pathological pain. As a key signaling of neurons-microglia interactions, the involvement of CX3CL1/CX3CR1 signaling in pathological pain has also been investigated extensively. The present study examined whether CX3CL1/CX3CR1 signaling plays a role in spinal LTP. The results showed that 10-trains tetanic stimulation (100 Hz, 2s) of the sciatic nerve (TSS) produced a significant LTP of C-fiber-evoked field potentials lasting for over 3 h in the rat spinal dorsal horn. Blockade of CX3CL1/CX3CR1 signaling with an anti-CX3CR1 neutralizing antibody (CX3CR1 AB) markedly suppressed TSS-induced LTP. Exogenous CX3CL1 significantly potentiated 3-trains TSS-induced LTP in rats. Consistently, spinal LTP of C-fiber-evoked field potentials was also induced by TSS (100 Hz, 1s, 4 trains) in all C57BL/6 wild type (WT) mice. However, in CX3CR1^-/-^ mice, TSS failed to induce LTP and behavioral hypersensitivity, confirming an essential role of CX3CR1 in spinal LTP induction. Furthermore, blockade of IL-18 or IL-23, the potential downstream factors of CX3CL1/CX3CR1 signaling, with IL-18 BP or anti-IL-23 neutralizing antibody (IL-23 AB), obviously suppressed spinal LTP in rats. These results suggest that CX3CL1/CX3CR1 signaling is involved in LTP of C-fiber-evoked field potentials in the rodent spinal dorsal horn.

## Introduction

As a cellular model of central sensitization in the spinal cord, spinal long-term potentiation (LTP) has been widely studied for exploring the mechanism of pathological pain [1–3]. Spinal LTP of C-fiber-evoked field potentials is usually induced *in vivo* by tetanic stimulation of the sciatic nerve (TSS) [1, 2], by which, a long-lasting allodynia, a common symptom of neuropathic pain, is also induced [4, 5]. Studies over the past decade indicate that lots of neuronal factors are involved in spinal LTP [6], such as N-methyl-D-aspartic acid (NMDA) receptor [7],neurokinin ½(NK)receptor [8], G protein-coupled metabotropic glutamate receptors (mGluRs) 1/5 [9] and Ca^2+^/calmodulin-dependent protein kinases II (CaMK II) [10]. The recent studies suggest that glial factors also contribute to spinal LTP [11], for instance, P2X4 receptors, p38 mitogen-activated protein kinase (p38 MAPK), P2X7 receptors, interleukin 1beta (IL-1beta) and tumor necrosis factor alpha (TNF-alpha) [12–15].

CX3CL1, a chemokine, has two functional forms: membrane-anchored CX3CL1 and soluble CX3CL1 [16], which is released from membrane by lysosomal cysteine protease Cathepsin S (Cat S) [17] or disintegrin and metalloproteinase (ADAM) 10/17[18, 19]. In the central nerve system, CX3CL1 is produced mostly in neurons, and its sole receptor CX3CR1, a G protein-coupled receptor, is mainly expressed in microglia [20–22]. Therefore, interaction between neurons and microglia may be mediated via CX3CL1/CX3CR1 signaling [20]. Increasing evidence suggests that spinal CX3CL1/CX3CR1 signaling plays a key role in the development and maintenance of pathological pain [23–27]. To address whether CX3CL1/CX3CR1 signaling is involved in central sensitization in the spinal cord, the present study was designed to illustrate the influence of CX3CL1/CX3CR1 signaling on spinal LTP.

## Materials and Methods

### Animal

Male adult Sprague Dawley rats (200–300 g, n = 128) were supplied by Shanghai Experimental Animal Center of the Chinese Academy of Sciences. C57BL/6NTac-[KO] CX3CR1 mice were purchased from Taconic Farms Inc. [28], and C57BL/6 background wild type (WT) control mice (male, 8–10 weeks) were purchased from The Jackson Laboratory and bred in the Animal Center of Fudan University. All animals were housed in a 12 h light/dark cycle with a room temperature of 22±1°C, and received food and water *ad libitum*. All experimental protocols and animal handling procedures were permitted by the Shanghai Animal Care and Use Committee, and were in line with the policies issued by the International Association for the Study of Pain.

### Immunohistochemical staining

After anesthetized with urethane, rats were perfused intracardially with warm saline followed with 4% cold paraformaldehyde in 0.1 M phosphate buffer (PB, pH 7.4). Thereafter, the L4–L6 segments of spinal cord were removed and postfixed in the same fixative for 2–4 h, then replaced with 10–30% gradient sucrose in PB for 24–48h at 4°C. The spinal cord tissues were transected into 35 μm sections by a freezing microtome (Leica, Germany). The sections were firstly blocked with 10% donkey serum in 0.3% Triton X-100 for 2 h at room temperature (RT), and then incubated for 24–72h at 4°C with a mixture of goat anti-CX3CL1 (1:500, R&D Systerms, USA), rabbit anti-CX3CR1 (1:2000, Torrey Pines Biolabs, USA), goat anti-IL-18 (1:500, R&D Systerms, USA), goat-anti-IL-18R (1:500, R&D Systerms, USA), mouse anti-GFAP (1:2000, Cell Signal Technology, USA), rabbit anti-IL-23 P19 (1:50, ABcam, Hong Kong), mouse anti-Neun (1:2000, Chemicon, USA), goat anti-Iba1 (1:500, ABcam, Hong Kong) or rabbit-anti-Iba1 (1:500, Wako, Japan). Subsequently, the sections were incubated with a mixture of rhodamine- and fluorescein isothiocyanate (FITC)-conjugated secondary antibodies (1:200; Jackson Immunoresearch, West Grove, PA, USA) for 2 hours at RT. The stained sections were examined by a confocal laser-scanning microscope (FV1000; Olympus, Tokyo, Japan).

### Electrophysiological recording of spinal LTP

The procedures were improved from the previous study [29]. Briefly, rats or mice were anesthetized with urethane (1.5g/kg, i.p.) for surgery. For drug injection, monitoring blood pressure and artificial ventilation, the right external jugular vein, carotid artery and the trachea were cannulated respectively. A laminectomy was performed at vertebrae T13-L1 to expose the lumbar enlargement of the spinal cord, and arachnoidea was incised and retracted longitudinally. The exposed tissue was covered with warm agar (2%), except the spinal cord column that was continually bathed in a pool of warm saline (37°C). The left sciatic nerve was exposed to delivery stimulation using bipolar silver electrodes, and covered with warm paraffin oil. Following electrical stimulation of the sciatic nerve, the field potentials were recorded in the ipsilateral L4–5 spinal cord segments with glass microelectrodes (impedance 3–6 MΩ), 300–800 μm in rats or 100–300 μm in mice from the surface of the cord. After recording stable responses following test stimuli (2x C-fiber threshold, 0.5 ms, 1.5-min interval) for > 40 min, conditioning tetanic stimulation (rats: 5x C-fiber threshold, 100 Hz, 10 trains of 2-s duration at 10-s interval; mice: 5x C-fiber threshold, 100 Hz, 4 trains of 1-s duration at 10-s interval) was delivered to the sciatic nerve for induced LTP of C-fiber-evoked field potentials. As a control, the sham group was not applied with conditioning tetanic stimulation. The signal was amplified by a microelectrode AC amplifier (A-M System, USA), and then recorded by CED systems (A/D converter Micro 1401 mk II, recording software Spike 2, CED, UK). Rabbit anti-CX3CR1 (Torrey Pines Biolabs, USA), recombinant mouse IL-18 BP (R&D Systerms, USA), recombinant rat CX3CL1 (R&D Systerms, USA), normal rabbit IgG (R&D Systerms, USA), goat IgG (Santa Cruz Biotechnology, USA) or 0.01M PBS was directly delivered to the surface of spinal cord in a volume of 30 μl.

### Electrophysiological recording procedures

Experiment 1: To show LTP of spinal C-fiber-evoked field potentials can be induced by 10-trains tetanic stimulation of the sciatic nerve (TSS), two groups of naïve adult male Sprague–Dawley rats were used to be applied with and without 10-trains TSS respectively. (TSS, n = 6; sham, n = 5).

Experiment 2: To test whether CX3CR1 is involved in TSS-induced LTP of spinal C-fiber-evoked field potentials, rats were divided into 2 groups: the anti-CX3CR1 antibody group (30 μg/30 μl, n = 7) and the control IgG group (30 μg/30 μl, n = 7). Anti-CX3CR1 antibody or IgG was applied 2h before delivering 10-trains TSS.

Experiment 3: Firstly, to avoid potential ceiling effect of 10-trains TSS on the rat spinal LTP, 3-trains TSS (n = 7) was used to induce a LTP with smaller potentiated extent than that of 10-trains TSS-induced LTP (n = 8). Thereafter, to examine whether 3-trains TSS-induced LTP can be potentiated by exogenous CX3CL1, exogenous CX3CL1 (0.75 μg/30 μl, n = 6) was administrated 30 min before 3-trains TSS. To address whether the effect of CX3CL1 was due to activation of CX3CR1, anti-CX3CR1 antibody (30 μg/30 μl, n = 6) or the control IgG (30 μg/30 μl, n = 6) was applied 1.5 h before delivering CX3CL1 (2 h before 3-trains TSS). Finally, the effect of CX3CL1 on baseline C-response was also examined by delivering exogenous CX3CL1 (3.75 μg/30 μl, n = 4; 0.75 μg/30 μl, n = 4) or PBS (30 μl, n = 4).

Experiment 4: The contributions of IL-18 and IL-23, the potential downstream factors of CX3CR1/CX3CL1, to spinal LTP were also examined in rats. We administrated IL-18 BP (7.0 μg/30 μl, n = 7; PBS 30 μl as the control, n = 8) 20 min before 10-trains TSS and anti-IL-23 antibody (6.0 μg/30 μl, n = 5; IgG 6.0 μg/30 μl as the control, n = 6) 40 min before 10-trains TSS to block the functions of IL-18 and IL-23, respectively.

Experiment 5: To confirm whether TSS induces LTP of spinal C-fiber-evoked field potentials in lack of CX3CR1 mice, knock-out mice lacking Cx3cr1 (CX3CR1^-/-,^ n = 4) and C57BL/6 background wild type control mice (n = 5) were used to be applied with 4-trains TSS.

### Cerebrospinal fluid and tissue collection

After defined survival times, rats were sacrificed by overdose of urethane and the L4–L6 spinal dorsal horn was rapidly removed. The dorsal horn tissues were homogenized with ultrasonic cell processor in an SDS sample buffer that contained a mixture of proteinase inhibitors and PMSF. To collect cerebrospinal fluid (CSF), a catheter (PE-10 tube) was inserted through the gap between the L4 and L5 vertebrae and extended to the subarachnoid space under sodium pentobarbital anesthesia (80 mg/kg, i.p.) and sterilizing. The CSF flowed out spontaneously through the catheter, when the rat body was erected.

### Western blots

Equal amount of protein or CSF sample was loaded and separated in 10% Tris-Tricine SDS–PAGE gel and transferred to PVDF membrane (Millipore). The membranes were blocked with 5% nonfat milk in Tris-buffered saline (pH 7.5) with 0.1% Tween-20 for 2 h at room temperature (RT), and incubated overnight at 4°C with goat anti-CX3CL1 antibody (1:500, R&D Systerms, USA), rabbit anti-CX3CR1 antibody (1:2000, Torrey Pines Biolabs, USA) or goat anti-Cathepsin S antibody (1:500, ABcam, Hong Kong). The blots were then incubated with HRP-conjugated secondary antibodies (1:1000, Pierce) for 2 h at RT. Signals were finally detected using enhanced chemiluminescence (ECL, Thermo, USA), and the bands were visualized with the ChemiDoc XRS system (Bio-Rad, USA). All Western blot analysis was performed at least three times, and consistent results were obtained.

Experiment 1: To test the effect of anti-CX3CR1 antibody (CX3CR1 AB) on the expression of CX3CR1, the spinal dorsal horn tissues were removed from rats of sham group (n = 4) in Experiment 1 of Electrophysiological recording, CX3CR1 AB group (n = 4) and IgG group (n = 4) in Experiment 2 of Electrophysiological recording, at the end of electrophysiological recording (3 h after TSS).

Experiment 2: To examine the expression of CX3CL1 after TSS, the spinal dorsal horn tissues and CSF were removed from rats applied with 10-trains TSS (0.5 h after TSS, n = 4) or sham (n = 4).

### ELISA Assay

To determine soluble CX3CL1 expression in CSF after TSS, we collected the spinal CSF (n = 6) from rats applied with 10-trains TSS (0.5 h after TSS) or sham. A rat CX3CL1 “Sandwich” ELISA kit (RayBiotech, USA) was used to examine CX3CL1 content the CSF. Rat recombinant FKN standards and samples in 100 μl were run in duplicate according to the manufacturer’s instructions. The optical density of each well was read at 450 nm. Data are expressed as percentage of FKN content in the basal fractions.

### Behavioral tests

The mechanical threshold was measured by probing *von* Frey filaments (Stoelting, USA). Each mouse was placed in a chamber (10cm×10cm×20cm) with customized platform that contains 1.5 mm diameter holes in a 5 mm grid of perpendicular rows throughout the entire area of the platform. Mice were allowed to acclimate for approximately 30min. A series of *von* Frey filament stimuli (0.16, 0.4, 0.6, 1.0, 1.4, 2.0g) were delivered to the central region of the plantar surface of the hindpaw with increasing bending force until the mouse withdrew the foot. Each filament was applied five times and each time maintained for 2s with 15s intervals. When the hindpaw withdrew from a filament at least three of the five applications, the value of the filament in grams was considered to be the“paw withdrawal threshold” (PWT).

The thermal threshold was measured by Hargreavestest. Mice were placed individually in transparent plastic chambers on an elevated glass surface. After acclimation to the test chambers for about 30min, a radiant heat source (IITC/Life Science Instruments) was focused on the hindpaw. The heat source was turned off when the mouse lifted the foot. The time from the onset of radiant heat application to withdrawal of the hindpaw was defined as the hindpaw withdrawl latency (PWL). To prevent tissue damage, the cut-off latency was set at 15s. The average of three trials was determined and the interval between trials is 10 min.

Hargreaves’test and *von* Frey test were performed before and 4 days after TSS in the same groups of CX3CR1^-/-^ (n = 8) and C57BL/6 WT (n = 8) mcie, started with *von* Frey test followed by Hargreaves’ test with an interval of 2 hours.

### Data analysis

All the data were expressed as means ± SEM. Student’s t-test (for comparisons of two groups) or One- (or two-) way ANOVA (for multiple group comparisons) followed by post hoc Student-Newmann-Keuls test was used to identify significant differences. In all cases, p < 0.05 was considered as being statistically significant.

## Results

### Expression of CX3CL1 and CX3CR1 in the spinal dorsal horn

**Fig 1:**
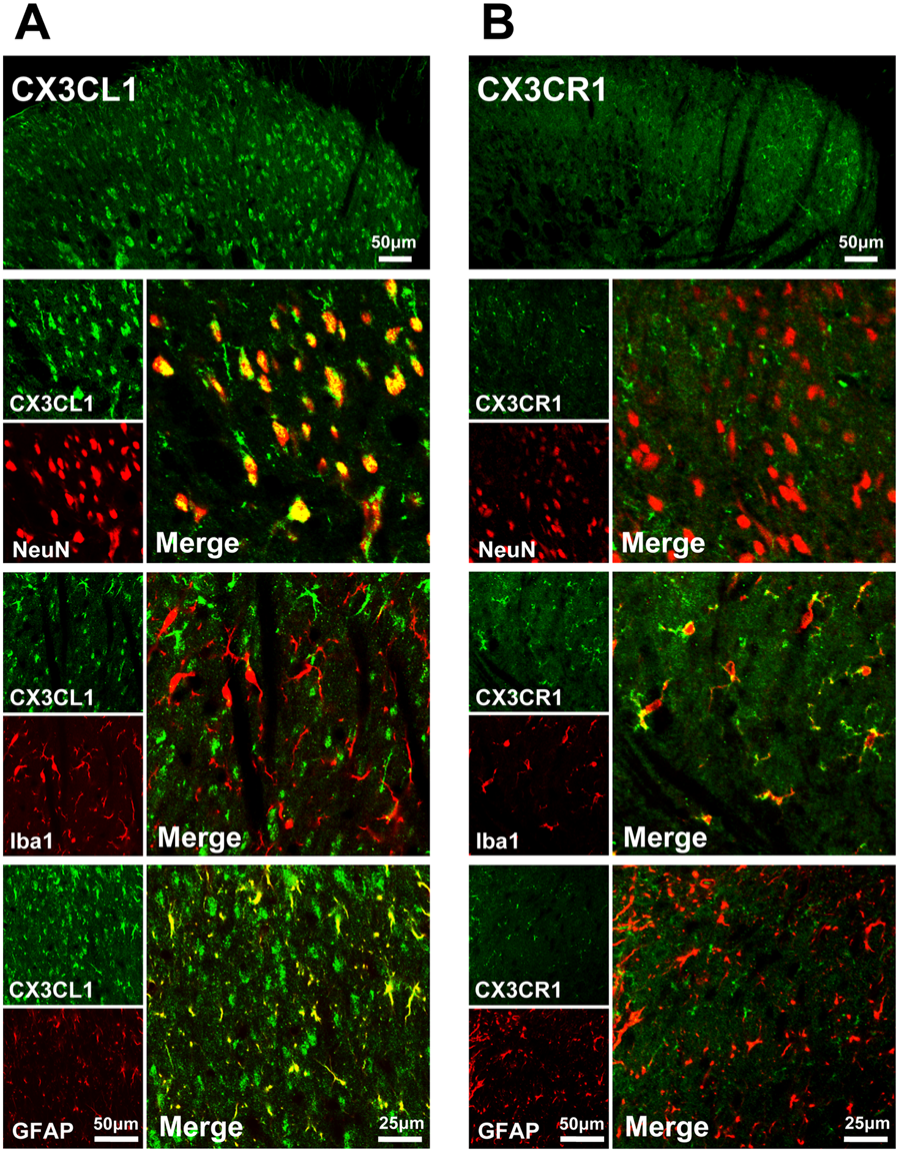
Expression of CX3CL1 and CX3CR1 in the spinal dorsal horn. Double immunofluorescence reveals that CX3CL1 co-localized with NeuN (neuronal marker) and GFAP (astrocyte marker), no immunoreactive singal in Iba1-labled microglia (A); CX3CR1 was expressed in Iba1-labled microglia in naïve rats and failed to co-localize with NeuN and GFAP (B).

Double immunostaining was performed on sections of the L4–6 spinal cord in rats. The distributions of CX3CL1 and CX3CR1 were examined in the spinal dorsal horn of naïve rats. Consistent with previous reports [21, 30], CX3CL1 was mostly expressed in NeuN (neuron marker)-labeled neuron and slightly in GFAP (astrocytic marker)-labeled cells in the spinal cord (Fig. 1A). Its receptor, CX3CR1 was mainly colocalized with Iba1 (microglia marker) (Fig. 1B).

### Blockade or knockout of CX3CR1 impairs spinal LTP

**Fig 2:**
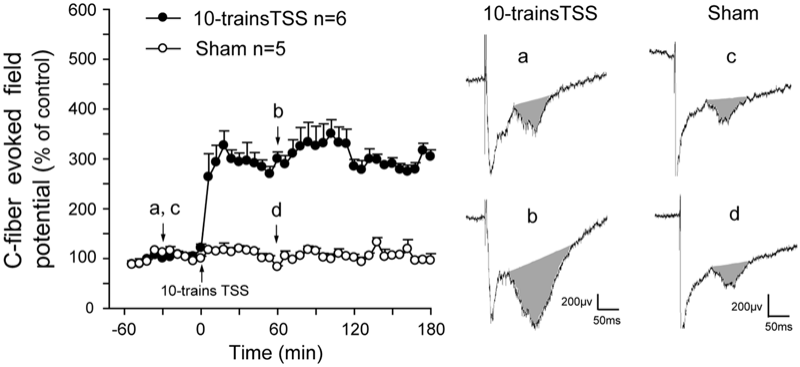
A representative spinal long-term potentiation (LTP) of C-fiber-evoked field potentials. Spinal LTP of C-fiber-evoked field potentials was induced by 10-trains tetanic stimulation of the sciatic nerve (TSS); conversely, it was not formed in the sham group (no TSS applied). a & b, the representative C-responses (gray area) in TSS group; c & d, the representative C-responses (gray area) in the sham group.

As described in our previous studies [5, 15, 31], tetanic stimulation of the sciatic nerve (TSS) produced a significant long-term potentiation (LTP) of C-fiber-evoked field potentials lasting for over 3 h in the rat spinal dorsal horn. The representative LTP was illustrated in Fig. 2. After 10-trains TSS, the C-fiber-evoked field potential was amplified about 3 folds than that before TSS. In contrast, in the sham group without TSS, no obvious change in C-response was observed (Two-way ANOVA, treatments: F_1, 9_ = 138.261, p < 0.01) (Fig. 2).

**Fig 3:**
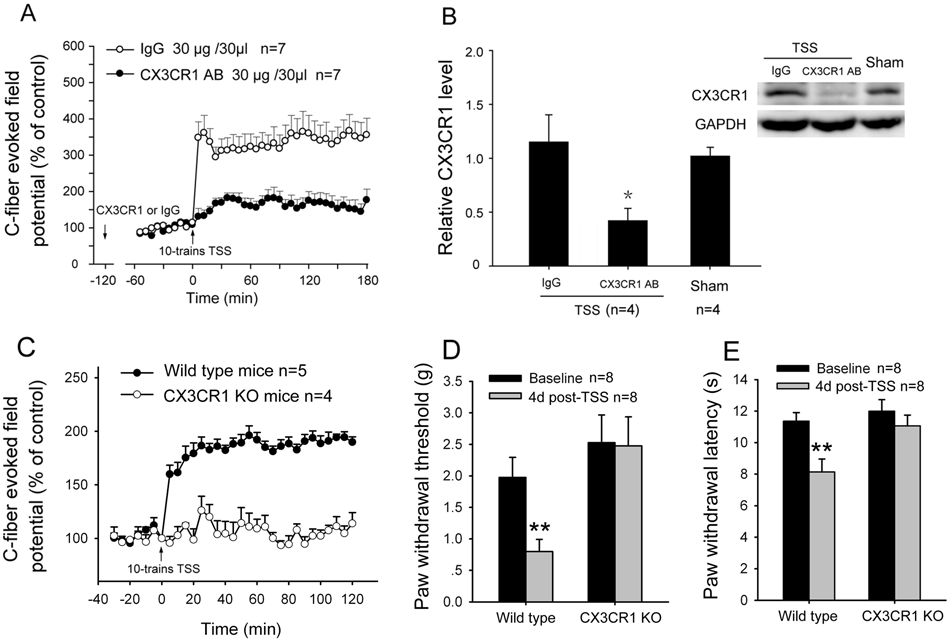
Involvement of CX3CR1 in spinal LTP. (A & B) When an anti-CX3CR1 neutralizing antibody (CX3CR1 AB, 30 μg/30 μl) was administrated 2 h before TSS, 10-trains TSS-induced spinal LTP was evidently suppressed (A). Simultaneously, the expression of CX3CR1 in spinal dorsal horn was also decreased by CX3CR1 AB (B). * p<0.05 vs. IgG control. (C) TSS stably induced spinal LTP of C-fiber-evoked field potentials in wild type mice, but failed to induce in CX3CL1 knockout mice. (D & E) TSS-induced mechanical allodynia (D) and thermal hyperalgesia (E) only occurred in wild type mice but not in CX3CL1 knockout mice. ** p>0.01 vs. Baseline (before TSS).

To examine whether CX3CL1/CX3CR1 signaling is involved in the spinal LTP, an anti-CX3CR1 neutralizing antibody (CX3CR1 AB)was applied to block CX3CL1/CX3CR1 signaling. As shown in Fig. 3A, the induction of spinal LTP was remarkably blocked by administration of CX3CR1 AB (30 μg/30 μl) 2h before 10-trains TSS, compared with control IgG (Two-way ANOVA, treatments: F_1, 12_ = 11.981, p<0.01). In addition, at the end of electrophysiological recording (3 h after TSS), the spinal dorsal horns were removed and the expression of CX3CR1 was examined by Western blots. Although no striking upregulation of CX3CR1 was observed after TSS, the expression of CX3CR1 was significantly decreased by delivering CX3CR1 AB, as compared with IgG (One-way ANOVA, F_2, 9_ = 5.399, p < 0.01) (Fig. 3B).

To further confirm CX3CL1/CX3CR1 signaling contributes to spinal LTP, TSS was delivered to the sciatic nerve for induced LTP of C-fiber-evoked field potentials in C57BL/6 WT and CX3CR1 knock-out mice. As described in the previous studies [32], spinal LTP of C-fiber-evoked field potentials was induced by a 4-trains TSS (100 Hz, 1s) in all five C57BL/6 WT mice, lasting for >2 h, with an amplitude increase of 92% at 1 h (Fig. 3C). Of note, LTP failed to be induced in CX3CR1 knock-out mice (Fig. 3C). Two-way ANOVA analysis revealed significant difference between groups (F_1, 16_ = 100.208, p<0.01). Combination with the results from rats and mice indicate an essential role of CX3CR1 in the induction of rodent spinal LTP.

Our previous studies showed that following TSS, a robust mechanical allodynia was observed in rats from day 1 after TSS and lasted at least for 7 days [4]. Blockade of CX3CR1 by anti-CX3CR1 antibody significantly suppressed TSS-induced mechanical allodynia [43]. In the present study, we further demonstrated that TSS, which conventionally induces LTP of C-fiber-evoked field potential in the WT mouse spinal dorsal horn, also produced a long-lasting mechanical allodynia and thermal hyperalgisa (Fig. 3D and 3E). Consistent with the electrophysiological results, behavioral tests showed that TSS-induced mechanical allodynia and thermal hyperalgesia did not occurred in CX3CR1 knock-out mice (Student’s t-test, p<0.01) (Fig. 3D-3F).

### CX3CL1 facilitates spinal LTP

**Fig 4:**
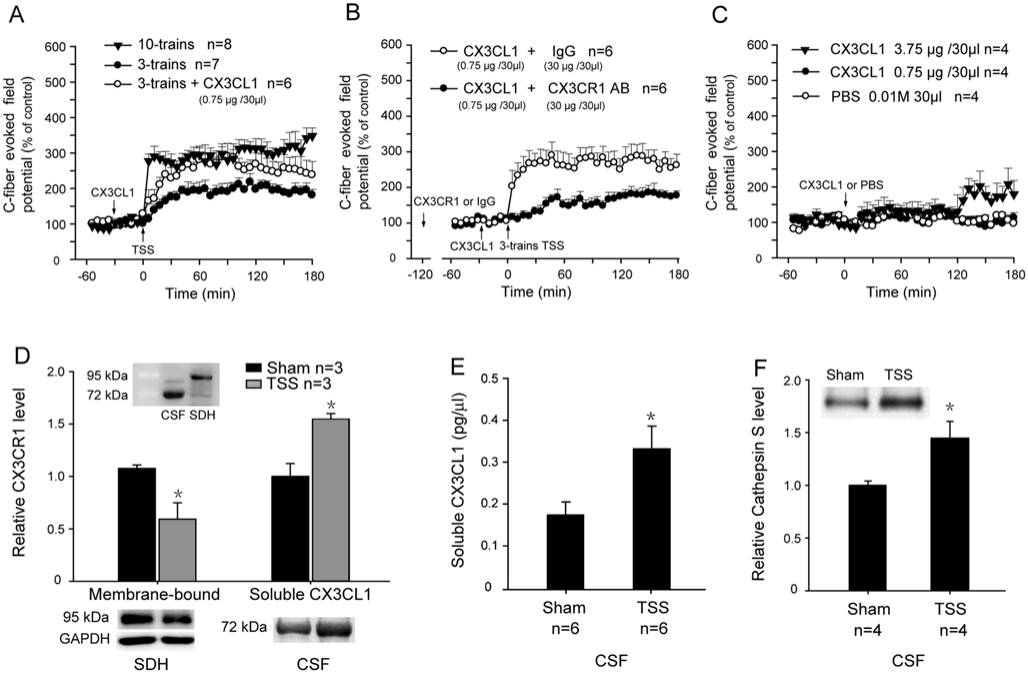
Involvement of CX3CL1 in spinal LTP. (A) As compared with 10-trains TSS-induced LTP, 3-trains TSS induced a LTP with smaller potentiated extent. While exogenous CX3CL1 (0.75 μg/30 μl) was applied 30 min before TSS, 3-trains TSS-induced LTP was robustly potentiated. (B) The facilitative effect of exogenous CX3CL1 (0.75 μg/30 μl) on 3 trains TSS-induced LTP was completely blocked by CX3CR1 AB (30 μg/30 μl), which was applied 2 h before TSS (1.5 h before delivering CX3CL1). (C) There was a delayed facilitative effect of 3.75 μg/30 μl exogenous CX3CL1 on baseline C-response, as compared with control PBS, and no influence of CX3CL1 was observed on baseline C-response at the dose of 0.75 μg/30 μl. (D) Western blot showed 30 min after 10-trains TSS, the expression of membrane-bound CX3CL1 was evidently reduced in the spinal dorsal horn, whereas soluble CX3CL1 level was upregulated in spinal CSF. Inset: the membrane-bound CX3CL1 and soluble CX3CL1 were detected at the 95 kDa and 72 kDa band respectively in the spinal dorsal horn (SDH) and CSF by an anti-CX3CL1 antibody. (E) ELASA assay showed that soluble CX3CL1 in the CSF was significantly upregulated at 30 min after TSS. (F) Western blot showed that Cathepsin S level was upregulated in the CSF at 30 min after TSS. * p<0.05 vs. Sham control.

To further verify the contribution of CX3CL1/CX3CR1 signaling to spinal LTP, exogenous CX3CL1 was applied to test whether LTP was facilitated. Considering spinal LTP could be saturated by strong stimulation of sciatic nerve [33], 3-trains TSS was used to induce spinal LTP to avoid the potential ceiling effect of 10-trains TSS on LTP in rats. The results showed that 3-trains TSS induced LTP with smaller potentiated extent than that of 10-trains TSS-induced LTP, and 3-trains TSS-induced LTP was robustly potentiated by spinal application of CX3CL1 (0.75 μg/30 μl) 30 min before 3-trains TSS (Two-way ANOVA, treatments: F_2, 18_ = 6.618, p < 0.01) (Fig. 4A).

To address whether the potentiated effect of CX3CL1 on 3-trains TSS-induced LTP was due to activation of CX3CR1, the effect of CX3CR1 AB on CX3CL1-induced enhancement of LTP amplitude was examined. Expectedly, application of 30 μg/30 μl CX3CR1 AB 2 h before 3-trains TSS (1.5 h before delivering CX3CL1), the potentiated effect of CX3CL1 (0.75 μg/30 μl) on 3-trains TSS-induced LTP was completely blocked (Two-way ANOVA, treatments: F_1, 10_ = 7.713, p < 0.05) (Fig. 4B). The results illustrated that the potentiated effect of CX3CL1 on spinal LTP was achieved via CX3CL1/CX3CR1 signaling. However, at the dose of 0.75 μg that markedly potentiated 3-trains TSS-induced LTP, CX3CL1 did not affect baseline C-response. Slight enhancement of basal C-response was only occurred at 2 h after application of a high dose (3.75 μg) CX3CL1 (Two-way ANOVA, treatments: F_2, 9_ = 2.263, p = 0.160) (Fig. 4C).

Several studies indicate that membrane-bound CX3CL1 is cleaved by the protease Cathepsin S (CatS), which is expressed and released by activated microglia [17, 34, 35]. Therefore, in the current work, whether soluble CX3CL1 was cleaved from neuronal membranes after TSS was examined. As shown in Fig. 4D, there were two bands detected by an anti-CX3CL1 antibody, a 95 kDa band predominantly expressed in the rat spinal dorsal horn (SDH) tissues and a 72 kDa band strongly expressed in the CSF (Fig. 4D inset), corresponding to membrane-bound CX3CL1 and soluble CX3CL1, respectively [25, 36, 37]. Following 10-trains TSS, membrane-bound CX3CL1 in the SDH was markedly reduced (Student t-test, t = 3.022, p < 0.05), whereas soluble CX3CL1 in the CSF was obviously increased at 30 min (Student t-test, t = 4.036, p < 0.05) (Fig. 4D). The soluble CX3CL1 in the CSF was further confirmed by ELISA assay (Fig. 4E). In addition, an increased protease Cathepsin S (Cat S) was also detected in the CSF 30 min after TSS (Student t-test, t = 2.720, p < 0.05) (Fig. 4F).

### IL-18 and IL-23 contributs to spinal LTP

**Fig 5:**
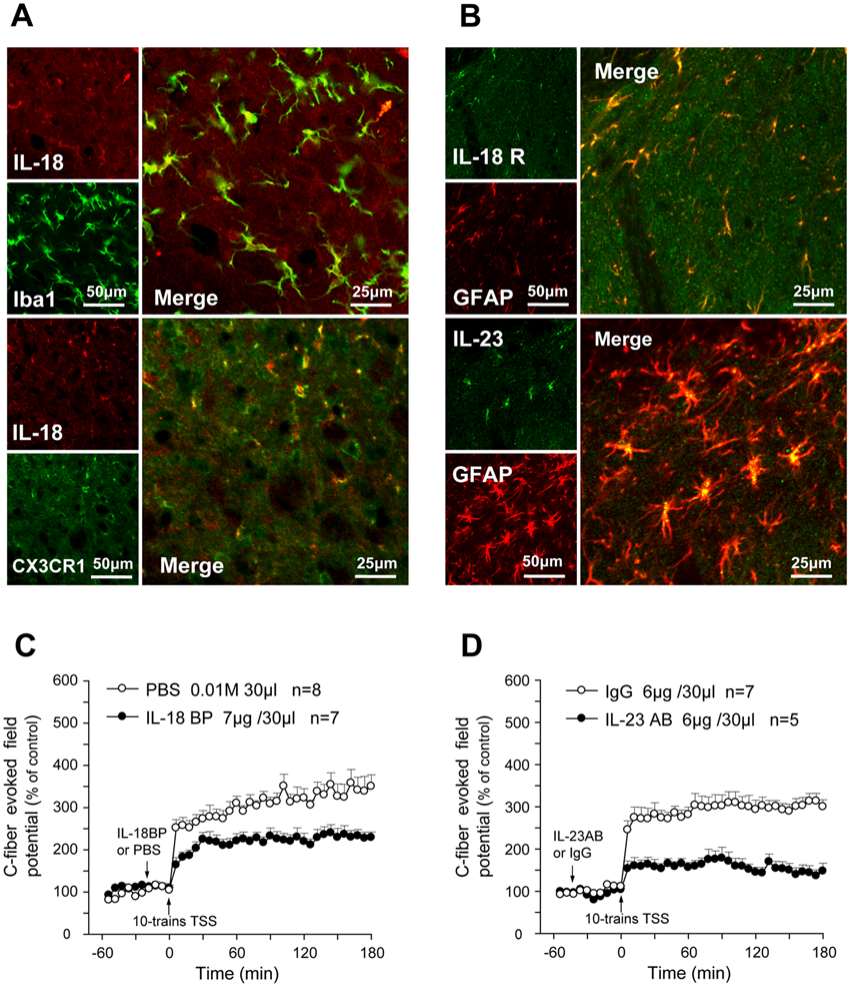
Contribution of IL-18 and IL-23 to spinal LTP. (A & B) In the spinal cord, IL-18 was mainly produced in Iba1-labled microglia and co-localized with CX3CR1 (A); both IL-18R and IL-23 were expressed in astrocytes (B). (C) As compared with control (PBS, 0.01M 30 μl), the spinal LTP was obviously suppressed by administrating IL-18BP (7 μg/30 μl) 20 min before 10-trains TSS. (D) Similarly, the spinal LTP was also suppressed by an anti-IL-23 neutralizing antibody (IL-23 AB, 6 μg/30 μl), which was administrated 40 min before 10-trains TSS.

Double immunostaining showed that in the spinal cord CX3CR1 colocalized with IL-18 that predominately expressed in spinal microglia in rats. In addition, IL-18 receptor and IL-23 were both mainly expressed in astrocytes in the spinal dorsal horn (Fig. 5A and B). We therefore examined the influences of IL-18 and IL-23 on rat spinal LTP. As shown in Fig. 5C and D, Spinal application of IL-18 BP (7.0 μg/30 μl) or anti-IL-23 antibody (IL-23 AB, 6.0 μg/30 μl) for 20 min (IL-18BP) or 40 min (IL-23 AB) before TSS significantly suppressed the spinal LTP of C-fiber-evoked field potential (Two-way ANOVA, IL-18BP treatments: F_1, 13_ = 10.485, p < 0.01; IL-23AB treatments: F_1, 10_ = 21.741) (Fig. 5C, D).

## Discussion

Unmyelinated C-fibers predominantly terminate in the superficial laminae of the spinal dorsal horn and mainly transfer nociceptive information. It is proved that the sensitization of unmyelinated C-fibers is the peripheral substrate of pathological pain [38–41]. C-fiber-evoked field potentials reflect the activation of pain-sensitive neurons in the superficial spinal dorsal horn. Long-term potentiation (LTP) of C-fiber-evoked field potentials is a phenomenon of central sensitization in the spinal cord, contributing to the development of pathological pain [2, 42]. It is showed that acute nerve injury can evoke both pathological pain and spinal LTP of C-fiber-evoked field potentials [31]. Compelling evidence has confirmed that tetanic stimulation of the sciatic nerve (TSS) not only evoked LTP of C-fiber-evoked field potentials, but also induced a long-lasting allodynia and hyperalgesia, the common symptom of neuropathic pain [4, 5, 43]. Accordingly, the investigation of spinal LTP of C-fiber-evoked field potentials will help us to understand the central mechanism underlying pathological pain.

Over the past decades, lots of neuronal factors were demonstrated to be involved in spinal LTP [6]. In recent years, the contribution of spinal glia to spinal LTP has also been focused on, and several glial factors have been considered to participate in spinal LTP, such as P2X4 receptors and p38 mitogen-activated protein kinase (p38 MAPK) [14], interleuk-1beta (IL-1beta) [15], tumor necrosis factor alpha (TNF-alpha) and P2X7 receptors [12, 13]. In the present study, another spinal microglial factor, CX3CL1/CX3CR1 signaling, was also involved in long-term potentiation (LTP) of C-fiber-evoked field potentials in the spinal dorsal horn.

Increasing evidence suggests that the activation of spinal glia plays an essential role in the development and maintenance of pathological pain [44, 45]. As a molecular model of central sensitization in the spinal cord [1–3], spinal long-term potentiation (LTP) has also been showed to be related with the activation of spinal glia [11, 12, 14, 15]. CX3CR1, a G protein-coupled receptor and the sole receptor of CX3CL1, is mainly expressed in spinal microglia [20, 21]. Binding with CX3CL1, microglia can be activated through p38MAPK signaling [25, 27], ERK1/2 signaling [46] and ERK5 signaling [47]. In addition, it has been demonstrated that CX3CL1/CX3CR1 signaling activity in spinal microglia is an essential process for development and maintenance of inflammatory pain [48, 49], neuropathic pain [25, 47] and cancer pain [27]. In line with such reports, the present findings of contribution of CX3CL1/CX3CR1 signaling to spinal LTP presents new evidence that CX3CL1/CX3CR1 signaling is involved in the potentiation of nociceptive transmission under the pathological pain condition.

CX3CL1 exists two functional forms: either membrane-bound or as a soluble glycoprotein [16]. The soluble form CX3CL1 performs chemoattractant activity for T cells and monocytes whilst membrane-bound CX3CL1 acts as an adhesion molecule contributing to leukocyte capture [16, 50]. The studies from Clark et al. showed that the levels of soluble CX3CL1 in CSF increased significantly after peripheral nerve injury, and lysosomal cysteine protease Cathepsin S played a key role in the release of soluble CX3CL1 from neuron membrane to CSF [17]. On the other hand, exogenous Cathepsin S-induced hyperalgesia and allodynia were attenuated by a neutralizing antibody against CX3CL1 [35]. Therefore, under pathological pain conditions, soluble CX3CL1 may be the main functional form, which is cleaved from neuronal membranes to activate the microglia via CX3CR1 and then contributes to amplification and maintenance of pathological pain. Although we did not observe the upregulation of CX3CR1 in the spinal dorsal horn at 3 h after TSS, another work from our laboratory showed that significant upregulation of CX3CR1 in the spinal cord occurred at 24 hours after TSS [51]. It is suggested that TSS-induced de novo synthesis of CX3CR1 may take more than 3 h. Interestingly, TSS induced an increased soluble CX3CL1 release, which may play an essential role in the enhanced CX3CL1/CX3CR1 signaling during spinal LTP.

In the current study, we also found the contribution of IL-18 and IL-23 to spinal LTP. In the spinal cord, IL-18 was considered to be a key modulator in pathological pain [52–54] and mediated microglia/astrocyte interaction [53]. Miyoshi et al. reported that the production of IL-18 in the spinal cord was regulated by p38MAPK [53]. On the other hand, exposing to exogenous CX3CL1, the p38MAPK signaling was activated in spinal microglia [25]. Consequently, it is reasonable to infer that CX3CR1 may be the upstream regulator of IL-18 in microglia.

As to IL-23, its role in the pathogenesis of multiple sclerosis (MS) has been studied [55–57]. However, the acquaintance with involvement of IL-23 in pathological pain remains limited. In the injured sciatic nerve of a mouse chronic constriction injury (CCI) model, the upregulation of IL-23 mRNA was observed [58]. In the current study, the finding of the involvement of IL-23 in spinal LTP provided direct evidence that spinal IL-23 may contribute to the potentiation of nociceptive transmission. The previous studies manifested that there are NF-kappa-B binding sites in p19 subunit gene promoter of IL-23, by binding with which NF-kappa-B could regulate IL-23 expression [59–61]. It was also found that NF-kappa-B could be activated in spinal IL-18R-expressing astrocytes after nerve injury, and the IL-18-induced allodynia was dose-dependently alleviated by intrathecal injection of an NF-kappa-B inhibitor, SN50, suggesting that nerve injury induces NF-kappa-B activation in the spinal astrocytes via the IL-18 signaling [53]. Accordingly, IL-23 may be regulated through IL-18/NF-kappa-B signaling. Therefore, it is conceivable that there may be a CX3CL1/IL-18/IL-23 signaling pathway contributing to spinal LTP.

Contrary to our finding of the facilitated effect of CX3CL1 on spinal LTP, the inhibitory influence of CX3CL1 on neuron excitability and central sensitization was reported. In the *in vitro* studies of cultured microglia, it was observed that CX3CL1 suppressed the releases of pro-inflammatory cytokines from activated microglia, such as TNF-alpha, IL-1beta, nitric oxide (NO) and IL-6 [62–64]. Some studies on hippocampus showed that CX3CL1 reduced excitatory postsynaptic response [65–67] and impaired the induction of LTP [68]. With regard to the contradictory effect of CX3CL1 in the central nervous system, one possibility may be attributed to the different concentrations to be used. In the work of Mizutani et al, 0.03 nM CX3CL1 significantly reduced LPS (lipopolysaccharide)-induced phosphorylation of ERK1/2 and secretion of TNF-alpha and IL-6 in macrophages, however, 3nM CX3CL1 elevated the expression of IL-23, which subsequently upregulated the production of TNF-alpha and abolished suppressive effect of low concentration of CX3CL1 [69]. This phenomenon suggests that different doses of CX3CL1 may induce different intracellular signaling and then perform inverse effects. In addition, given that two novel functional isoforms of CX3CR1 have identified [68], it is possible that the different isoforms of CXCR1 exert contradictory functions via diverse signaling pathways [46].

In conclusion, the present study showed that CX3CL1/CX3CR1 signaling was involved in long-term potentiation (LTP) of C-fiber-evoked field potentials in the spinal dorsal horn.
